# Massively parallel sequencing analysis of synchronous fibroepithelial lesions supports the concept of progression from fibroadenoma to phyllodes tumor

**DOI:** 10.1038/npjbcancer.2016.35

**Published:** 2016-11-16

**Authors:** Salvatore Piscuoglio, Felipe C Geyer, Kathleen A Burke, Melissa P Murray, Charlotte KY Ng, Alba Mota, Caterina Marchio, Samuel H Berman, Larry Norton, Edi Brogi, Britta Weigelt, Jorge S Reis-Filho

**Affiliations:** 1 Department of Pathology, Memorial Sloan Kettering Cancer Center, New York, NY, USA; 2 Department of Pathology, Hospital Israelita Albert Einstein, Instituto Israelita de Ensino e Pesquisa, São Paulo, Brazil; 3 Departamento de Bioquímica, Universidad Autónoma de Madrid (UAM), Instituto de Investigaciones Biomédicas ‘Alberto Sols’ (CSIC-UAM), IdiPAZ, Madrid, Spain; 4 Department of Medical Sciences, University of Turin, Turin, Italy; 5 Department of Medicine, Memorial Sloan Kettering Cancer Center, New York, NY, USA

## Abstract

Phyllodes tumors (PTs) and fibroadenomas (FAs) are fibroepithelial lesions (FELs) of the breast. Although mutations affecting exon 2 of *MED12* are highly recurrent in FAs and PTs, *TERT* promoter hotspot mutations are frequently found in PTs but are vanishingly rare in FAs. Malignant transformation of benign PTs is well-documented, but the progression from FA to PT remains a matter of contention. Here we report on the somatic genetic alterations in multiple ipsilateral synchronous FELs (three FAs, one benign PT, and one malignant PT) occurring in the same patient. DNA samples extracted from each tumor and matched normal tissue were subjected to targeted massively parallel sequencing using the Memorial Sloan Kettering-Integrated Mutation Profiling of Actionable Cancer Targets (MSK-IMPACT) assay. This analysis revealed *MED12* mutations in all lesions. One FA and the benign PT harbored a *MED12*^Gly44Val^ mutation, whereas another FA and the malignant PT displayed a *MED12*^Gly44Asp^ mutation. The remaining FA had an independent distinct *MED12*^Gly44Cys^ mutation. A formal clonality analysis suggested a clonal relationship between the FELs with identical *MED12* mutations (*P*<0.05). A clonal *TERT* promoter hotspot mutation was identified exclusively in the malignant PT. The identification of distinct *MED12* mutations in multifocal ipsilateral and synchronous FELs supports the notion that co-existing mammary fibroepithelial tumors can arise independently. Conversely, the co-existence of identical *MED12* mutations indicates clonal relatedness among FAs and PTs, corroborating the hypothesis that FAs may constitute the substrate from which PTs develop. Our findings also support the notion that acquisition of *TERT* promoter mutations may drive the progression of FELs.

## Introduction

Mammary fibroepithelial lesions (FELs) include fibroadenomas (FAs) and phyllodes tumors (PTs).^1^ FAs account for the majority of FELs, have a benign behavior, and are not uncommonly multifocal. Histologically, FAs are characterized by an admixture of epithelial and stromal components arranged into pericanalicular and/or intracanalicular growth patterns.^1^ By contrast, PTs are relatively rare neoplasms, accounting for ~2.5% of all mammary FELs.^1^ Histologically, PTs are characterized by an overt intracanalicular growth pattern and a mesenchymal component with varying cellularity and atypia, resulting in the formation of leaf-like projections into epithelium-lined spaces.^1^ PTs are graded as benign, borderline, or malignant depending on the stromal features, including cellularity, nuclear atypia, and proliferation rate,^1^ and this grading system correlates, albeit imperfectly, with their clinical behavior. Although FAs are benign lesions with limited growth potential, PTs have a tendency for continuous growth, can recur locally regardless of grade, and metastasize.^1^ Metastatic events, however, are essentially limited to malignant PTs or occur after local recurrence with grade progression following a benign diagnosis.^2^

FAs and PTs have recently been shown to be underpinned by highly recurrent hotspot *MED12* exon 2 mutations.^3–9^ These mutations likely constitute founder events in the development of most FAs and a subset of PTs, but appear to be neither necessary nor sufficient to confer the more aggressive behavior characteristic of PTs.^3–10^ Recent genomic analyses of FAs and PTs^6,9^ demonstrated that consistent with the differences in morphologic appearance and clinical behavior, PTs are genetically more advanced and display a higher mutational burden than FAs.^9^ In addition to *MED12* mutations, FAs and PTs were found to harbor co-occurring *RARA* mutations.^6,9^ Although recurrent mutations in *FLNA*, *SETD2*, and *KMT2D* were found across the spectrum of PTs, mutations in *bona fide* cancer genes (e.g., *TP53* and *RB1*) were only detected in borderline and malignant PTs.^6,9^ Recurrent clonal hotspot mutations in the *TERT* promoter and *TERT* gene amplifications have also been documented in FELs.^4,6,11^ Although these *TERT* alterations have been detected in the majority of borderline and malignant PTs, they have also been detected in a small subset of benign PTs,^4,6,11^ and shown to be either specific for^6^ or enriched in^11^ PTs as compared with FAs.

Although there is evidence that benign and borderline PTs can progress to malignant PTs,^12^ the progression from FAs to PTs remains controversial, despite the not uncommon finding of histologically FA-like areas in PTs and the existence of molecular evidence based on the loss of heterozygosity and human androgen receptor assays, suggesting that some PTs may arise from FAs.^13,14^ Interestingly, the frequency of *MED12* mutations appears to diminish with increasing PT grade,^4–6^ whereas the frequency of the *TERT* −124C>T promoter hotspot mutation and/or *TERT* gene amplification has been shown to increase according to the histologic grade of PTs, with significantly lower rates in benign (18%) than in borderline (57%) and malignant PTs (68%).^6^ On the basis of these observations, we have posited that genetic alterations affecting *TERT* rather than *MED12* mutations may have a role in the progression of PTs.^6^

Given that the clonal relatedness analyses between FAs and PTs carried out to date may not be entirely conclusive, we have employed targeted massively parallel sequencing (MPS) to investigate the repertoire of somatic genetic alterations and the clonal relatedness of multifocal ipsilateral FELs in the breast of a female patient.

## Results

**Figure 1:**
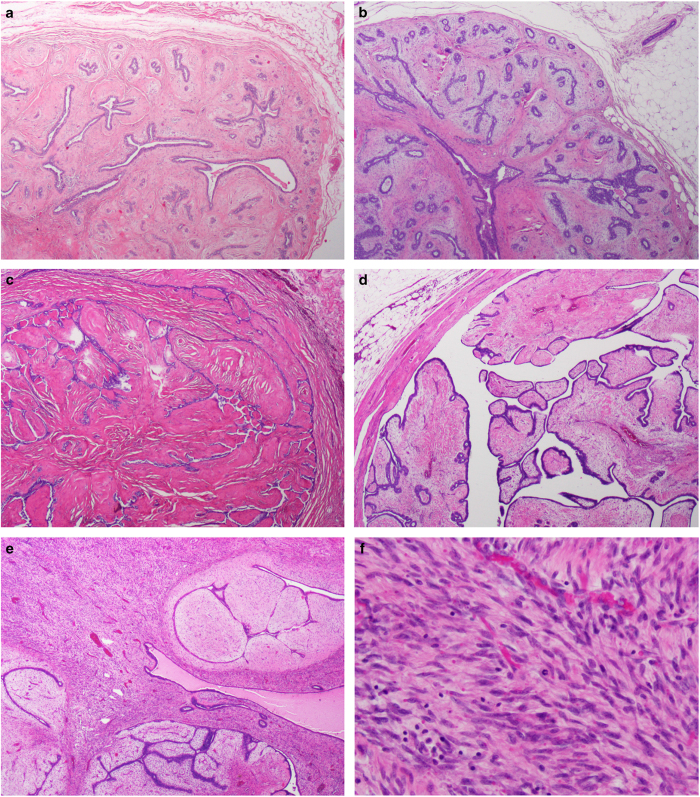
Representative micrographs of the ipsilateral synchronous mammary fibroepithelial lesions evaluated in this study. Representative micrographs of three fibroadenomas (FAs) (**a**–**c**), one benign phyllodes tumor (PT) (**d**), and one malignant PT (**e**). Two FAs (**a**,**b**) displayed a predominant pericanalicular pattern and a stromal component with usual cellularity, and no atypia or mitoses. The third FA had an intracanalicular growth pattern and hyalinized stroma (**c**). The benign and malignant PTs were characterized by leaf-like architecture (**d**,**e**). The benign PT showed moderate stromal cellularity, no significant nuclear pleomorphism, and inconspicuous mitotic activity (0 mitoses per 10 high-power fields (HPFs) (**d**); Supplementary Table 1), whereas the malignant PT displayed stromal expansion with marked hypercellularity (**e**), moderate nuclear atypia, and mitotic activity (10 mitoses per 10 HPFs (**f**); Supplementary Table 1). The epithelium did not show atypia nor did it show significant proliferation in all lesions.

A 37-year-old female patient was diagnosed with five synchronous and ipsilateral FELs in the right breast. A detailed description of the clinical history and gross analysis of mastectomy specimen is provided in the Supplementary Material and Supplementary Figure 1. After consensus histologic review by four pathologists with an interest in breast pathology (FCG, MPM, EB, and JSR-F), the lesions were classified as FAs (*n*=3), benign PT (*n*=1), and malignant PT (*n*=1) according to World Health Organization classification^1^ (Figure 1; Supplementary Table 1).

**Figure 2:**
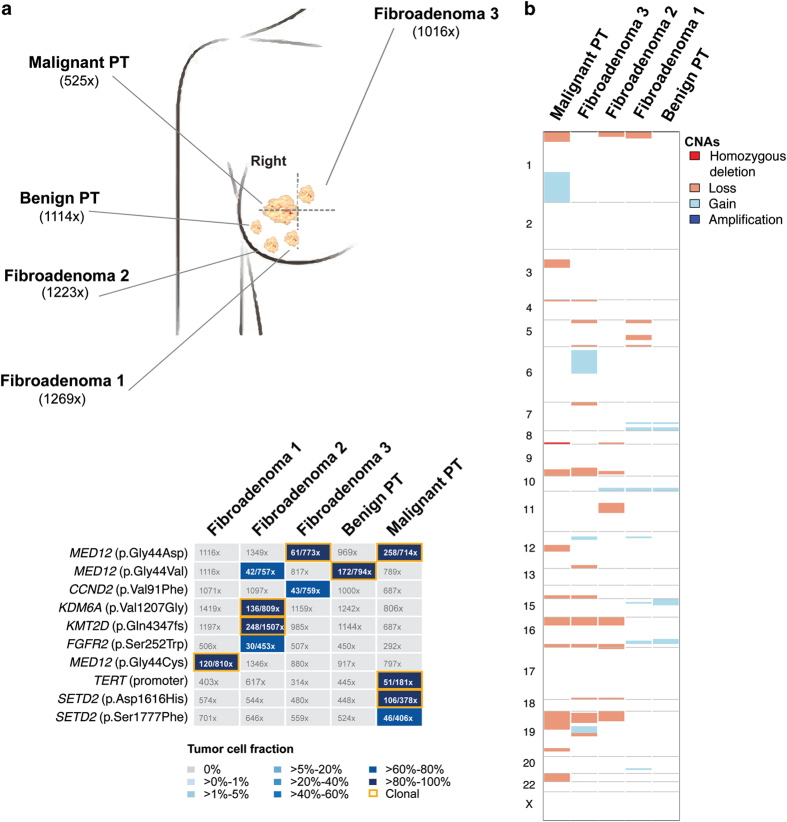
Targeted capture massively parallel sequencing (MPS) analysis of five synchronously diagnosed fibroepithelial lesions. (**a**) Schematic illustration of the anatomical location of the five synchronously diagnosed fibroepithelial tumors in the right breast of a 37-year-old woman (top panel) and the results of the targeted capture MPS analysis (bottom panel). Tumor cell fractions (TCFs) were based on an integrative analysis of mutant allele fractions, tumor cell content, ploidy, and local copy number using ABSOLUTE,^20^ and reported for the non-synonymous and promoter somatic mutations identified in a given lesion. TCFs are color-coded according to the legend. Clonal mutations are highlighted in orange boxes. In samples harboring a given mutation, the number of mutant alleles (numerator) detected is specified in its respective text box, over the depth of coverage for that specific locus (denominator). PT, phyllodes tumor. (**b**) Heatmap illustrates the repertoire of copy number alterations as defined by targeted capture MPS. Samples are represented as columns and chromosomes are represented along the *y* axis, with dark gray lines demarcating the chromosomes and the light gray dotted lines representing centromeres. Absolute copy number was defined by ABSOLUTE^20^ and are color-coded according to the legend to the right of the heatmap.

Representative formalin-fixed paraffin-embedded sections were microdissected, and DNA of each of the five lesions and of adjacent normal tissue was subjected to high-depth targeted MPS using the Memorial Sloan Kettering-Integrated Mutation Profiling of Actionable Cancer Targets (MSK-IMPACT) assay, targeting all coding regions and select non-coding and regulatory regions of 410 genes. MSK-IMPACT sequencing yielded a median coverage of 1,065× (range 389×–1,269×; Supplementary Table 2) and revealed a median of three non-synonymous somatic mutations per lesion (range 1–5; Supplementary Table 3). The five FELs displayed *MED12* hotspot mutations, which were clonal in all lesions except FA2 (Figure 2; Supplementary Table 3) and validated using Sanger sequencing and amplicon re-sequencing (Supplementary Figure 2; Supplementary Table 4). FA2 and the benign PT harbored an identical *MED12*^Gly44Val^ mutation, whereas FA3 and the malignant PT harbored an identical *MED12*^Gly44Asp^ mutation. The third FA displayed a distinct *MED12*^Gly44Cys^ mutation. A clonal relatedness analysis based on a previously reported approach^15^ revealed that the probability of two unrelated FELs sharing identical *MED12* mutations by chance was low (probabilities of identical Gly44Val (c.131G>T) and Gly44Asp (c.131G>A) by chance were 0.027 and 0.006, respectively, Supplementary Table 5), suggesting that the benign PT was clonally related to FA2, whereas the malignant PT was likely clonally related to FA3 (both *P*<0.05, see Materials and methods). As the mutational repertoire of FELs of the breast differs from that of common cancers, we performed additional Sanger sequencing analysis of all coding regions of the *FLNA* gene, which is among the genes most frequently mutated in FAs (5%) and PTs (87%) and is not included in the list of genes analyzed by MSK-IMPACT. None of the samples analyzed was found to harbor a somatic mutation affecting *FLNA*.

Given that clonal relatedness based on the presence of a single shared mutation, despite the statistically significant results, may be ambiguous, we sought to define whether the proposed clonal relatedness between FA2 and the benign PT, and between FA3 and the malignant PT would be supported by their pattern of copy number alterations (CNAs). In fact, copy number analysis revealed a similar pattern of CNAs in FA3 and the malignant PT, including losses of 4p, 9q, 16p, 16q, and 19p (Figure 2c; Supplementary Figure 3). In addition, FA3 and the malignant PT shared three non-telomeric and non-centromeric DNA breakpoints. DNA breakpoints have been shown to provide genetic information that is useful to define the clonal relationship between tumor samples.^16^ The partial identity score was calculated based on these DNA breakpoints, as previously described by Bollet *et al.*,^16^ for the comparisons between FA3 and the malignant PT as well as between 22 unrelated previously described PTs^6^ (Supplementary Methods). The upper limit of the 95% confidence interval of the comparisons between FAs and PTs from other patients was employed as a cutoff to infer whether two tumors were clonally related. This revealed a partial identity score between FA3 and the malignant PT of 0.1227, above the 0.0328 cutoff determined from the analysis of the samples included in this study and 22 unrelated PTs (median 0.007). Taken together, these findings support the clonal relatedness between FA3 and the malignant PT. On the other hand, CNA analysis was non-informative to infer clonal relatedness between FA2 and the benign PT, given that the benign PT displayed a flat profile with minimal CNAs (Figure 2c; Supplementary Figure 3).

Presumed pathogenic somatic mutations affecting *FGFR2*^Ser252Trp^, *KDM6A*^Val1207Gly^, and *KMT2D*^Gln4347fs^ were only found in FA2, whereas a clonal *TERT* promoter −124 C>T hotspot mutation and two distinct mutations in *SETD2* were found exclusively in the malignant PT (Figure 2a; Supplementary Figure 1; Supplementary Table 3), one of which was subclonal. Additional subclonal mutations were detected in the FELs analyzed (Figure 2a; Supplementary Table 3), including a subclonal *CCND2* mutation, which was present in FA3 but absent in its clonaly related malignant PT. These findings provide evidence to suggest that FELs may display intratumor genetic heterogeneity.

## Discussion

FAs and PTs share histologic similarities, however, their diagnoses carry fundamentally distinct clinical implications. Although FAs are benign and do not necessarily require surgical excision, PTs mandate surgical excision with clear margins and can display malignant behavior. Despite their distinctive natural history, progression from FAs to PTs has been previously hypothesized.^9,13,14,17,18^ Observational studies have also reported on the development of malignant PTs in patients with a previous history of FAs^18^ and the presence of FA-like areas in PTs,^9^ and studies based on loss of heterozygosity and human androgen receptor assays have suggested clonal relatedness between synchronous and metachronous FAs and PTs.^13,14^ Interestingly, patients with previous history of FAs have been shown to display better outcomes than those with *de novo* malignant PTs (i.e., without previous history of FA).^18^

Recent data demonstrating similar mutational profiles in both FAs and PTs with frequent co-occurrence of *MED12* and *RARA* mutations indirectly support the hypothesis that FAs and PTs may be clonally related. Upon analysis of five cases of concurrent or longitudinal FAs and PTs, Tan *et al.*^9^ found shared mutations in two cases, suggesting clonal relatedness. No formal clonality analysis was performed and potential drivers of progression to PTs were not identified.^9^ Here we provide additional molecular evidence that PTs (including a malignant PT) may originate from FAs based on the presence of identical somatic *MED12* mutations and similar pattern of CNAs and breakpoints. Our sequencing analysis also revealed that only the malignant PT harbored a *TERT* promoter mutation. We have recently described that *TERT* promoter hotspot mutations are exclusively found in PTs, and that their frequency increases with histologic grade;^6^ consistent with these observations, a *TERT* promoter hotspot mutation was solely found in the malignant PT and not in its clonally related FA. Hence, our findings reported here and in Piscuoglio *et al.*^6^ are consistent with the notion that *TERT* alterations may have a role in the progression to a malignant phenotype within FELs. Given that *TERT* promoter hotspot mutations (i.e., −124C>T and −146C>T) and *TERT* gene amplifications have been shown to result in increased levels of telomerase expression and, potentially, activity in somatic cells,^19^ allowing them to bypass the Hayflick limit (i.e., telomeric crisis) and to undergo a higher number of cell divisions, we posit that these alterations may allow for the mesenchymal cells of PTs to proliferate and acquire further mutations and/or CNAs, which may ultimately result in a more aggressive form of disease. Further studies are warranted to define the mechanistic basis of the progression from FAs to PTs and the role of *TERT* somatic genetic alterations in FELs of the breast.

The FELs studied here were anatomically separated (Supplementary Material), although some were found to be clonally related. A field effect is one plausible explanation for the clonal relatedness between FA2 and the benign PT, and between FA3 and the malignant PT. According to this theory, a single progenitor clone expands to populate widespread areas of an organ and may give rise to multifocal anatomically separated lesions after acquisition of independent genetic events. Therefore, it is possible that specialized mammary stromal cells (e.g., the likeliest cell of origin of FELs) may acquire *MED12* mutations, populate large areas of the breast, and subsequently give rise to distinct FELs. In fact, although FA3 and the malignant PT shared an identical *MED12* mutation and multiple DNA breakpoints, they harbored private genetic alterations, including a *CCND2* somatic mutation in FA3 and somatic mutations affecting *SETD2* and the promoter of *TERT* in the malignant PT. These findings are consistent with the hypothesis that these two lesions shared a common ancestor, but diverged early in their tumor evolution.

This study has several limitations. First, we have studied samples from a single patient. Despite this limitation, the clonal relatedness analysis performed here and the results reported by Tan *et al.*^9^ are in agreement in that FAs may be clonally related to PTs, and that distinct FELs in the same patient may harbor distinct *MED12* mutations. Second, MSK-IMPACT yielded a limited number of somatic mutations in FAs, thus whole-exome or whole-genome sequencing analysis could theoretically have provided more conclusive results. It should be noted, however, that the number of genes mutated beyond *MED12* in FAs has been shown to be limited,^9^ rendering the additional information provided by whole-exome sequencing likely non-informative for clonality analysis. As the samples included in this study were formalin-fixed paraffin-embedded, whole-genome sequencing could not be performed. Despite these limitations, our data support the contention that a subset of FAs is clonal, neoplastic, and may constitute the substrate from which some PTs arise. Although our data support the contention that a subset of FAs are precursors of benign, as well as malignant PTs, we do not advocate a change in the current treatment of FAs, given that the likelihood of and time for the progression from FAs to PTs has yet to be fully defined. On the basis of the prevalence of FAs, it is plausible that the progression rate of FAs to PTs is likely to be sufficiently low to allow FAs to be followed rather than surgically excised, unless other clinicopathological variables indicate excision.

## Materials and methods

### Case

The diagnostic slides and formalin-fixed paraffin-embedded tissue blocks of five synchronous ipsilateral mammary FELs from a 37-year-old female patient were retrieved from the Department of Pathology of Memorial Sloan Kettering Cancer Center (MSKCC). The samples were anonymized before analysis, the study was approved by the MSKCC Institutional Review Board (WA047-14 and WA0102-13), and informed consent was obtained. All histologic sections of the FELs were independently reviewed by four expert breast pathologists (FCG, MPM, EB, and JSR-F) and classified according to the latest World Health Organization criteria.^1^ Microdissection and nucleic acid extraction were performed as previously described^6^ (Supplementary Methods).

### Targeted capture MPS

MPS analyses of the DNA extracted from the five FELs and the matched normal were profiled using the MSK-IMPACT sequencing assay using validated protocols^6,15^ (Supplementary Methods).

The tumor cell fraction of each mutation, in a way akin to the cancer cell fraction, and the absolute copy number were inferred using ABSOLUTE (v1.0.6)^20^ as previously described^6,21^ (Supplementary Methods).

### *FLNA* gene Sanger sequencing

Sanger sequencing of all coding regions of *FLNA* was performed in all five samples, as previously described.^6^ Details of the sequencing analysis and primer sequences are available in the Supplementary Methods and Supplementary Table 6.

### Validation of mutations identified by MPS using Sanger sequencing and amplicon resequencing

Somatic mutations in *MED12* (exon 2) and *TERT* promoter were validated using Sanger sequencing and amplicon resequencing, as previously described.^6,21^ Details of the sequencing analysis and primer sequences are available in the Supplementary Methods and Supplementary Table 5.

### Clonal relatedness of synchronously diagnosed FELs

To determine whether the five FELs were clonally related, we used the previously published frequency of *MED12* exon 2 mutations in 98 unrelated FAs^3^ and 79 unrelated PTs^9^ to determine the probability of two unrelated FELs sharing identical *MED12* exon 2 mutations,^3,9^ adopting a previously described approach.^15^ On the basis of these datasets, the Gly44Val (c.131G>T) and Gly44Asp (c.131G>A) mutations were found in 8% (14/177) and 16% (29/177) of FAs and PTs, respectively, and the probabilities of two unrelated samples sharing identical Gly44Val (c.131G>T) and Gly44Asp (c.131G>A) were 0.006 and 0.027, respectively (Supplementary Table 5). Given the reported prevalence of *MED12* mutations varies across the various publications, we further applied the same approach to a data set from our previous publications consisting of 100 unrelated FAs^6^ and 76 unrelated PTs,^6^ with 7% (13/176) and 21% (37/176) harboring the Gly44Val (c.131G>T) and Gly44Asp (c.131G>A) mutations, respectively, with the probabilities of two unrelated samples sharing these identical mutations as 0.006 and 0.044, respectively (Supplementary Table 5).

### Availability of data and materials

Sequencing data have been deposited to the National Center for Biotechnology Information Sequence Read Archive under the accession SRP062618.
