# Supplementary material for: Massively parallel sequencing analysis of synchronous fibroepithelial lesions supports the concept of progression from fibroadenoma to phyllodes tumor
Source: NPJ Breast Cancer. 2016 Nov 16;2:16035–. doi: 10.1038/npjbcancer.2016.35 (PMC5515337; doi:10.1038/npjbcancer.2016.35)
Supplement: Supplementary Methods [file npjbcancer201635-s1.doc]

**SUPPLEMENTARY MATERIAL**

**Clinical history and gross analysis of mastectomy specimen**

A 37-year old patient presented at Memorial Sloan Kettering Cancer Center with a complaint of red and firm right breast, suggestive of inflammatory process or inflammatory breast cancer. Initial mammogram, however, showed a large mass occupying the lower and upper outer quadrants and measuring 10.0 cm at the largest dimension (Supplementary Figure 1A). Subsequent ultrasound confirmed the presence of a large nodule, but also revealed multiple satellite lesions, measuring up to 1.9 cm (Supplementary Figure 1B). A biopsy of the large mass was performed, which revealed a fibroepithelial lesion consistent with malignant PT. The patient underwent a total mastectomy. Gross analysis of the surgical specimen showed a multilobulated dominant mass measuring 10.5 x 10.0 x 7.9 cm, in addition to four peripheral circumscribed nodules. Among these satellite lesions, the largest (FA3) was located in the intersection of the upper quadrants, 1.8 cm superior to the dominant mass (malignant PT), and measured 1.9 x 1.3 x 1.1 cm. The three additional satellite lesions were located in the lower outer quadrant: one at 6/7 o’clock, 0.8 cm anteroinferior to the main mass and measuring 1.1 x 1.0 x 0.8 cm (FA1); one at 7 o’clock, 0,6 cm to the main mass, measuring 1.0 x 1.0 x 1.0 cm (FA2); and one at 8/9 o’clock, 3,0 cm antero-nferior to the main mass and 1.5 cm to FA2, measuring 0.7 x 0.7 x 0.7 cm (benign PT). Two blocks per cm of the dominant mass (malignant PT) and the entirety of the four satellite lesions were sampled for histological assessment.

**SUPPLEMENTARY METHODS**

**Microdissection and nucleic acid extraction**

Eight-µm-thick representative sections of each tumor and its respective normal tissue were stained with nuclear fast red and microdissected using a sterile needle under a stereomicroscope (Olympus SZ61) for the selection of normal tissue devoid of neoplastic cells, as previously described.^1^ DNA extraction and quantification was performed as previously described.^1^

**Targeted capture massively parallel sequencing**

MPS analyses of the DNA extracted from the three FAs, the one benign PT, the one malignant PT and the matched normal was performed using the MSK-IMPACT sequencing assay. This sequencing assay contains optimized baits for all coding regions of 410 genes and select intronic and regulatory elements of selected genes. MPS was performed on an Illumina HiSeq2500. Sequence alignment and processing, as well as the identification of somatic mutations and allele-specific copy number alterations (CNAs) were performed (CNAs) were performed using FACETS^2^ as previously described^3^. The breakpoints were determined using the output segmentation files from FACETS. Non-telomeric and non-centromeric segment ends were compared between FA3 and the malignant PT, segment ends that were within 50bp of each other were considered exact.

The tumor cell fraction (TCF, referred to as cancer cell fraction in the original publication^4^) of each mutation and the absolute copy number of CNAs were inferred using ABSOLUTE (v1.0.6)^4^ as previously described.^3^ Given that the allele fractions of somatic mutations as observed from MPS analyses are compound measures of tumor purity, tumor ploidy, local copy number, normal cell contamination and sequencing errors, ABSOLUTE can be used to infer the TCF of mutations and the absolute copy number to provide an intuitive way of interpreting the results. For this analysis, as previously described,^3^ the segmented Log_2_ ratios from copy number data and the mutant allele fractions were used as input for ABSOLUTE^4^, which jointly estimates the tumor purity and ploidy and resolves equivocal inferences using a large database of tissue-specific karyotypes^4^. ABSOLUTE solutions were manually reviewed as recommended.^4,5^ A mutation was classified as clonal if the inferred probability was >50%,^5^ or if the lower bound of the 95% confidence interval of its TCF was >90%. Mutations that did not meet the above criteria were considered subclonal.

Genome plots illustrating copy number profiles were scaled to equal tumor cell content based on the purity estimates from ABSOLUTE as previously described.^6^

***FLNA* Sanger Sequencing**

All coding regions (exons 2 to 47) of the *FLNA* gene, which is recurrently mutated in breast fibroepithelial lesions and not included in MSK-IMPACT assay, were Sanger sequenced. Primer sequences are provided in Supplementary Table 6. PCR amplification and Sanger sequencing were performed as previously described.^3^ Sequences of the forward and reverse strands were analyzed using MacVector software (MacVector, Inc, Cary, NC).^1^

**Validation of mutations identified by MPS using Sanger sequencing and amplicon resequencing**

Somatic mutations in *MED12* (exon 2) and *TERT* promoter were validated using Sanger sequencing and amplicon resequencing. Primer sequences are described in Piscuoglio et al.^3^ PCR primer sets that amplify these loci were described elsewere.^1^ PCR amplification and Sanger sequencing were performed as previously described.^3^ Sequences of the forward and reverse strands were analyzed using MacVector software (MacVector, Inc, Cary, NC).^1^ All analyses were performed in triplicate. Amplicon resequencing was performed on fragments amplified with primers as described in Piscuoglio et al.^3^ Pooled amplicons were sequenced on an Illumina MiSeq using the 150×150 chemistry and analyzed as previously described.^3^

**Analysis of clonal relatedness based on *MED12* mutation data**

To determine the clonal relatedness of two FELs, we used a previously published approach to determine the probability of observing a given mutation in both samples by chance^7^. Briefly, given two samples, the probability of observing a given mutation in both samples is given by the binomial probability , *n*=2, *k*=2, and *p* is defined as the frequency of the mutation in the prior datasets of FELs. Thus the probability of observing a given set of *M* identical mutations in the 2 samples is given by
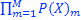
. The previously published datasets by independent investigators (98 fibroadenomas, Lim et al.^8^ and 79 phyllodes tumors, Tan et al.^9^) and our group (100 fibroadenomas and 76 phyllodes tumors^10^) were employed to determine the prevalence of *MED12* exon 2 mutations in FELs. The probability of two FELs sharing a given mutation was calculated based on the prevalence of Gly44Val(c.131G>T) and Gly44Asp(c.131G>A) mutations in the 98 fibroadenomas^8^, 79 phyllodes tumors^9^, 177 fibroadenomas and phyllodes tumors^8,9^, 100 fibroadenomas^10^, 76 phyllodes tumors^10^ and 176 fibroadenomas and phyllodes tumors^10^. For the cohort from our own group, we further excluded the five samples that are included in the current study (i.e. three fibroadenomas, one benign phyllodes tumor and one malignant phyllodes tumor), resulting in 97 fibroadenomas, 74 phyllodes tumors, and 171 fibroadenomas and phyllodes tumors. In all scenarios except for the Gly44Asp (c.131G>A) mutation based on the 100 fibroadenomas^10^ or the 97 fibroadenomas, the probability of sharing either Gly44Val(c.131G>T) or Gly44Asp(c.131G>A) was <0.05 (Supplementary Table 5).

**Analysis of clonal relatedness based on DNA breakpoints**

To determine clonal relatedness among the five fibroepithelial lesions studied, the breakpoints were determined using the output segmentation files from FACETS^2^. Non-telomeric and non-centromeric segment ends were compared between FA3 and the malignant PT, segment ends that were within 50bp of each other were considered exact. The breakpoints were then analyzed using the equation for calculating the partial identity score (PS), as previously described by Bollet et al.^11^. To define the PSs between two unrelated lesions, we tested each of the fibroepithelial lesions included in this study and 22 previously reported PTs from distinct patients^12^. This analysis revealed a median PS of 0.007 (95% confidence interval 0 – 0.0328) for FELs from distinct patients. The confidence interval for the control samples was determined using the bootstrapping, using the 'bootstrap' cran package. Boot strapping was performed based on the sample median for 1000 iterations, and the 95% confidence interval was calculated using bootstrap percentile method. The upper limit of the 95% confidence interval was then used as a PS cutoff for identifying tumors that are clonally related.

**SUPPLEMENTARY REFERENCES**

1 Weinreb, I. *et al.* Hotspot activating PRKD1 somatic mutations in polymorphous low-grade adenocarcinomas of the salivary glands. *Nat Genet* **46**, 1166-1169, doi:10.1038/ng.3096 (2014).

2 Shen, R. & Seshan, V. E. FACETS: allele-specific copy number and clonal heterogeneity analysis tool for high-throughput DNA sequencing. *Nucleic Acids Res*, doi:10.1093/nar/gkw520 (2016).

3 Guerini-Rocco, E. *et al.* Microglandular adenosis associated with triple-negative breast cancer is a neoplastic lesion of triple-negative phenotype harbouring TP53 somatic mutations. *The Journal of pathology* **238**, 677-688, doi:10.1002/path.4691 (2016).

4 Carter, S. L. *et al.* Absolute quantification of somatic DNA alterations in human cancer. *Nature biotechnology* **30**, 413-421, doi:10.1038/nbt.2203 (2012).

5 Landau, D. A. *et al.* Evolution and impact of subclonal mutations in chronic lymphocytic leukemia. *Cell* **152**, 714-726, doi:10.1016/j.cell.2013.01.019 (2013).

6 van de Wiel, M. A. *et al.* CGHcall: calling aberrations for array CGH tumor profiles. *Bioinformatics* **23**, 892-894, doi:10.1093/bioinformatics/btm030 (2007).

7 Schultheis, A. M. *et al.* Massively Parallel Sequencing-Based Clonality Analysis of Synchronous Endometrioid Endometrial and Ovarian Carcinomas. *Journal of the National Cancer Institute* **108**, pii: djv427, doi:10.1093/jnci/djv427 (2016).

8 Lim, W. K. *et al.* Exome sequencing identifies highly recurrent MED12 somatic mutations in breast fibroadenoma. *Nature genetics* **46**, 877-880, doi:10.1038/ng.3037 (2014).

9 Tan, E. Y. *et al.* Recurrent phyllodes tumours of the breast: pathological features and clinical implications. *ANZ journal of surgery* **76**, 476-480, doi:10.1111/j.1445-2197.2006.03754.x (2006).

10 Piscuoglio, S. *et al.* Massively parallel sequencing of phyllodes tumours of the breast reveals actionable mutations, and TERT promoter hotspot mutations and TERT gene amplification as likely drivers of progression. *The Journal of pathology* **238**, 508-518, doi:10.1002/path.4672 (2016).

11 Bollet, M. A. *et al.* High-resolution mapping of DNA breakpoints to define true recurrences among ipsilateral breast cancers. *Journal of the National Cancer Institute* **100**, 48-58, doi:10.1093/jnci/djm266 (2008).

12 Piscuoglio, S. *et al.* MED12 somatic mutations in fibroadenomas and phyllodes tumours of the breast. *Histopathology* **67**, 719-729, doi:10.1111/his.12712 (2015).
