# Supplementary figures and images for: Massively parallel sequencing analysis of synchronous fibroepithelial lesions supports the concept of progression from fibroadenoma to phyllodes tumor
Source: NPJ Breast Cancer. 2016 Nov 16;2:16035–. doi: 10.1038/npjbcancer.2016.35 (PMC5515337; doi:10.1038/npjbcancer.2016.35)

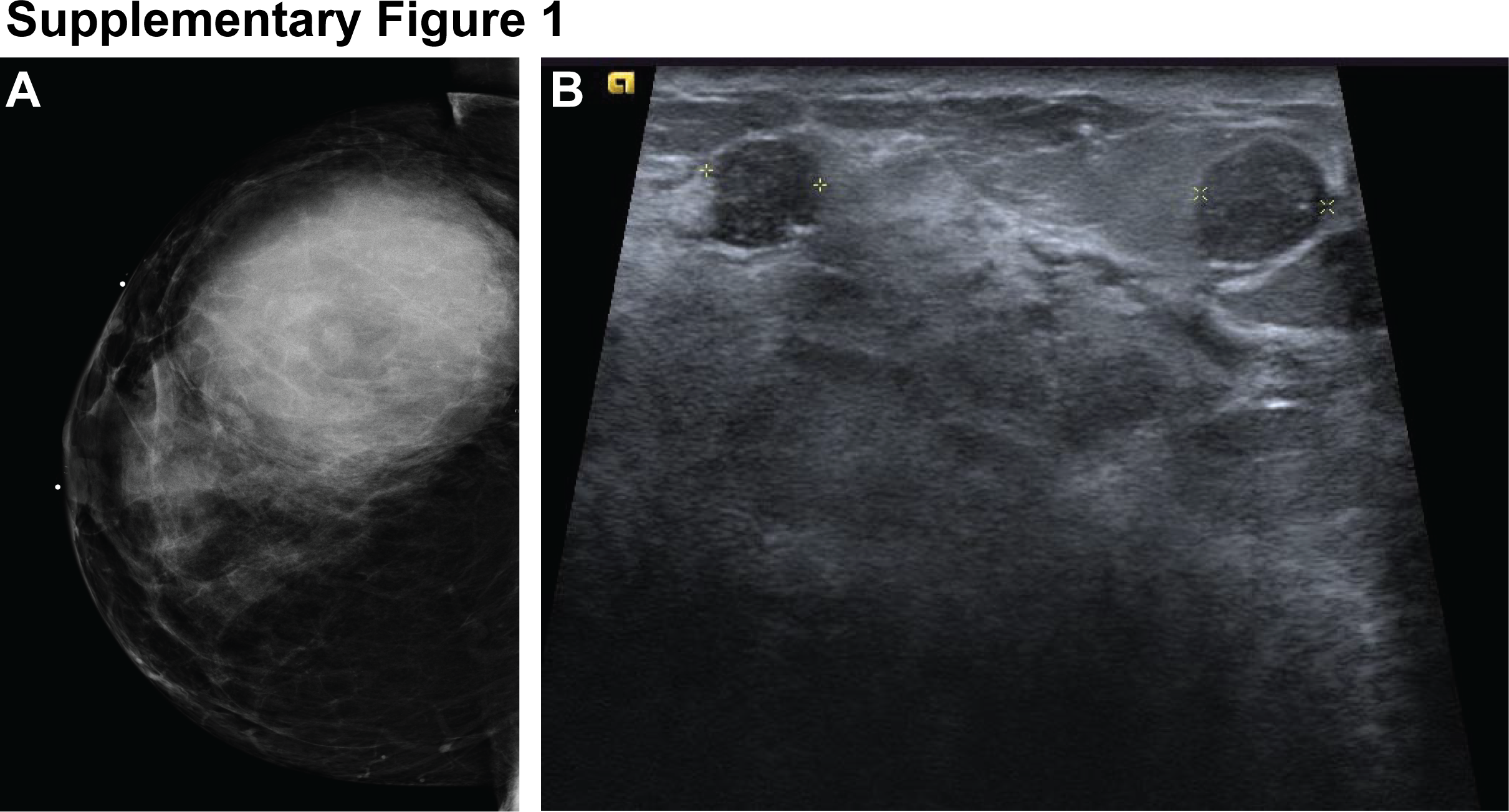

Supplement: Supplementary Figure S1 [file npjbcancer201635-s2.tiff]

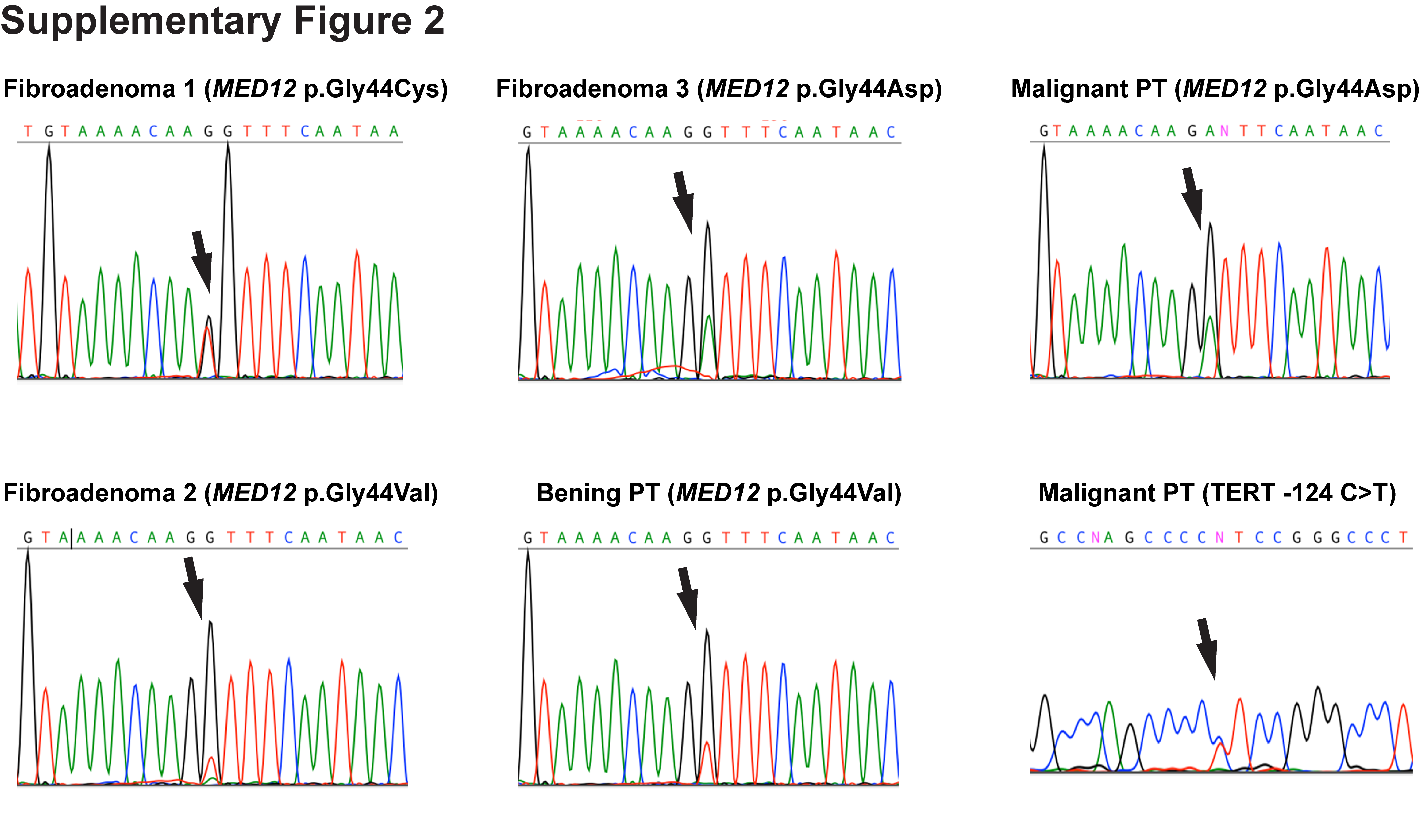

Supplement: Supplementary Figure S2 [file npjbcancer201635-s3.tiff]

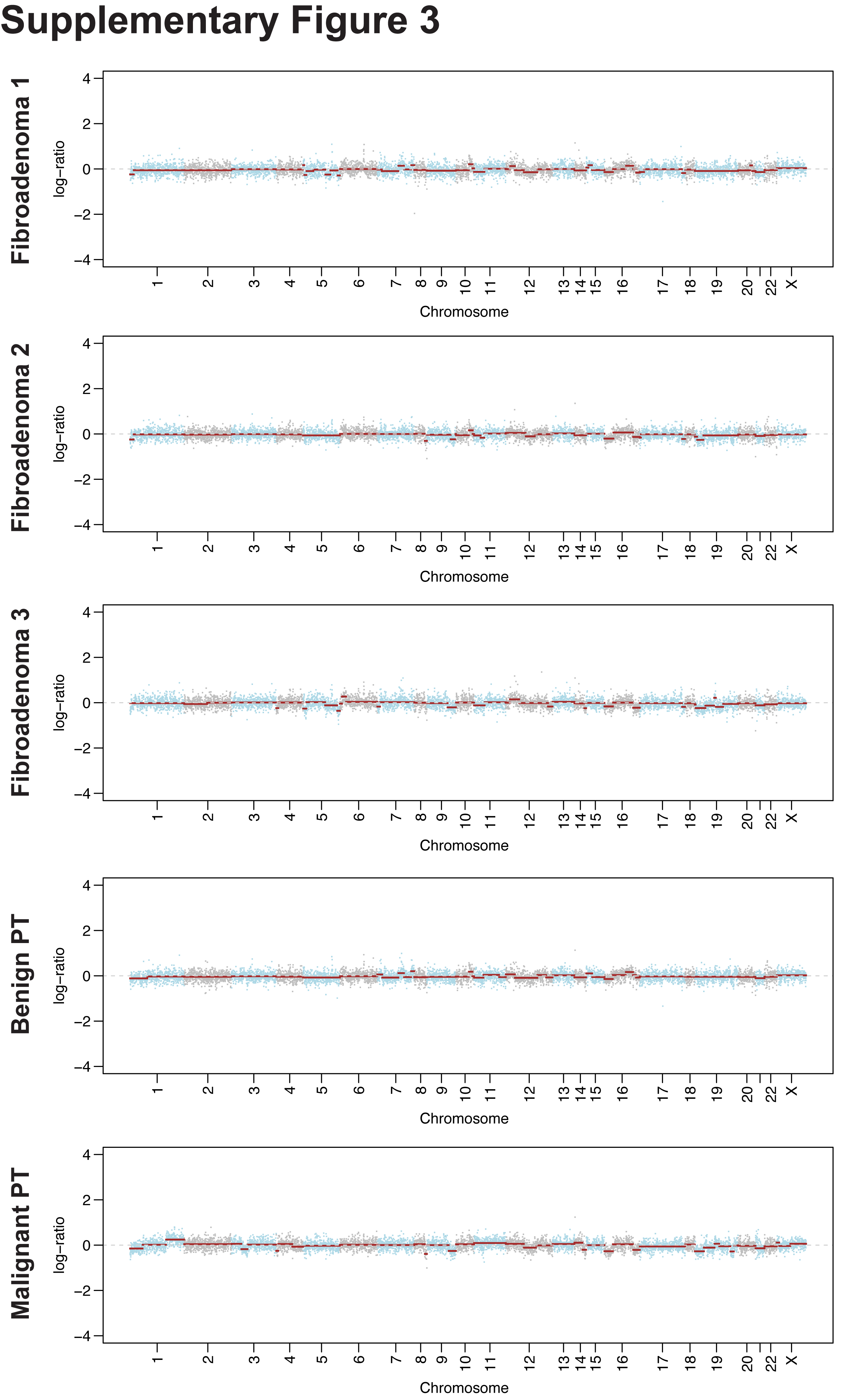

Supplement: Supplementary Figure S3 [file npjbcancer201635-s4.tiff]
