# Supplementary material for: Massively parallel sequencing analysis of synchronous fibroepithelial lesions supports the concept of progression from fibroadenoma to phyllodes tumor
Source: NPJ Breast Cancer. 2016 Nov 16;2:16035–. doi: 10.1038/npjbcancer.2016.35 (PMC5515337; doi:10.1038/npjbcancer.2016.35)
Supplement: Supplementary Table 1 [file npjbcancer201635-s5.pdf]

Supplementary Table 1: Clinico-pathologic characteristics of the lesions included in this study, and sequencing analyses performed.

Sample ID	Tumor border	Stromal cellularity	Stromal atypia	Mitotic activity per 10 HPFs	Stromal overgrowth	Malignant heterologous elements	MSK-IMPACT	Sanger Sequencing	Amplicon re-sequencing
**Benign PT**	Well defined	Moderate	Mild	0	Absent	Absent	Y	Y	Y
**Malignant PT**	Infiltrative	Moderate to marked	Moderate	10	Absent	Absent	Y	Y	Y
**Fibroadenoma 1**	Well defined	Mild	Mild	0	Absent	Absent	Y	Y	Y
**Fibroadenoma 2**	Well defined	Mild	Mild	0	Absent	Absent	Y	Y	Y
**Fibroadenoma 3**	Well defined	Mild	Mild	0	Absent	Absent	Y	Y	Y

PT: Phyllodes tumor.
