# Supplementary material for: Massively parallel sequencing analysis of synchronous fibroepithelial lesions supports the concept of progression from fibroadenoma to phyllodes tumor
Source: NPJ Breast Cancer. 2016 Nov 16;2:16035–. doi: 10.1038/npjbcancer.2016.35 (PMC5515337; doi:10.1038/npjbcancer.2016.35)
Supplement: Supplementary Table 2 [file npjbcancer201635-s6.pdf]

Supplementary Table 2: Targeted capture massively parallel sequencing statistics.

Sample ID	Total Reads	Mean Target Coverage	% Target Bases 2X	% Target Bases 10X	% Target Bases 20X	% Target Bases 30X	% Target Bases 40X	% Target Bases 50X	% Target Bases 100X
Fibroadenoma 1	55,653,134	1268.7	99.41%	99.26%	99.17%	99.10%	99.05%	99.00%	98.73%
Fibroadenoma 2	61,530,625	1222.5	99.39%	99.22%	99.13%	99.07%	99.02%	98.95%	98.64%
Fibroadenoma 3	48,431,217	1016.3	99.44%	99.28%	99.18%	99.11%	99.06%	99.01%	98.67%
Benign PT	49,667,209	1113.5	99.38%	99.22%	99.12%	99.07%	99.01%	98.94%	98.60%
Germline	17,456,475	397.7	99.25%	99.00%	98.77%	98.57%	98.38%	98.17%	96.13%
Malignant PT	34,085,474	754.3	99.36%	99.13%	99.02%	98.92%	98.80%	98.69%	98.12%
