# Supplementary material for: Massively parallel sequencing analysis of synchronous fibroepithelial lesions supports the concept of progression from fibroadenoma to phyllodes tumor
Source: NPJ Breast Cancer. 2016 Nov 16;2:16035–. doi: 10.1038/npjbcancer.2016.35 (PMC5515337; doi:10.1038/npjbcancer.2016.35)
Supplement: Supplementary Table 3 [file npjbcancer201635-s7.pdf]

Supplementary Table 3: List of somatic mutations found in the fibroepithelial lesions of the breast subjected to targeted capture massively parallel sequencing.

Sample ID	Gene	Amino Acid Change	Effect	MAF	Depth	LOH Status	Tumor Cell Fraction (TCF) (ABSOLUTE)	Probability of mutation being clonal	Lower bound of 95% confidence interval	Clonal/ Subclonal mutation	Normal Depth	Chromosome	Position	Reference Allele	Alternate Allele	Mutation Taster	CHASM (Breast)	FATHMM	PROVEAN (in-frame indels only)	Kandoth et al 127 significantly mutated genes	Lawrence et al Cancer5000-S	Cancer Gene Census	Pathogenicity
Benign PT	*MED12*	p.Gly44Val	missense_variant	21.66%	794	No LOH	1.00	0.9925	0.9900	Clonal	482	X	70339254	G	T	D	.	PASSENGER/OTHER			TRUE	TRUE	Non-pathogenic
Fibroadenoma 1	*MED12*	p.Gly44Cys	missense_variant	14.81%	810	No LOH	0.99	0.8618	0.8145	Clonal	482	X	70339253	G	T	D	.	PASSENGER/OTHER			TRUE	TRUE	Non-pathogenic
Fibroadenoma 2	*FGFR2*	p.Ser252Trp	missense_variant	6.62%	453	No LOH	0.80	0.4870	0.5561	Subclonal	142	10	123279677	G	C	A	Driver	CANCER		TRUE	TRUE	TRUE	Likely pathogenic
Fibroadenoma 2	*KDM6A*	p.Val1207Gly	missense_variant	16.81%	809	No LOH	1.00	0.9768	0.9529	Clonal	388	X	44945140	T	G	D	Driver	PASSENGER/OTHER		TRUE	TRUE	TRUE	Likely pathogenic
Fibroadenoma 2	*KMT2D*	p.Gln4347fs	frameshift_variant	16.46%	1507	No LOH	1.00	0.9833	0.9720	Clonal	604	12	49425446	CCT	C	.				TRUE	TRUE	TRUE	Likely pathogenic
Fibroadenoma 2	*MED12*	p.Gly44Val	missense_variant	5.55%	757	No LOH	0.67	0.0429	0.4947	Subclonal	482	X	70339254	G	T	D	Passenger	PASSENGER/OTHER			TRUE	TRUE	Non-pathogenic
Fibroadenoma 3	*CCND2*	p.Val91Phe	missense_variant	5.67%	759	No LOH	0.76	0.0002	0.6558	Subclonal	364	12	4385246	G	T	D	Passenger	PASSENGER/OTHER				TRUE	Non-pathogenic
Fibroadenoma 3	*MED12*	p.Gly44Asp	missense_variant	7.89%	773	No LOH	1.00	0.9445	0.9077	Clonal	482	X	70339254	G	A	D	Passenger	PASSENGER/OTHER			TRUE	TRUE	Non-pathogenic
Fibroadenoma 3	*PTPRT*	p.Arg459Arg	synonymous_variant	6.96%	704	No LOH	0.93	0.7116	0.8146	Clonal	236	20	41100979	C	T	.	.	.					Non-pathogenic
Malignant PT	*MED12*	p.Gly44Asp	missense_variant	36.10%	714	No LOH	1.00	0.7922	0.9779	Clonal	482	X	70339254	G	A	D	Passenger	PASSENGER/OTHER			TRUE	TRUE	Non-pathogenic
Malignant PT	*SETD2*	p.Asp1616His	missense_variant	28.00%	378	No LOH	1.00	0.9263	0.9375	Clonal	183	3	47144907	C	G	D	Passenger	PASSENGER/OTHER		TRUE	TRUE	TRUE	Non-pathogenic
Malignant PT	*SETD2*	p.Ser1777Phe	missense_variant	11.40%	406	No LOH	0.71	0.0746	0.5335	Subclonal	228	3	47127752	G	A	D	Passenger	PASSENGER/OTHER		TRUE	TRUE	TRUE	Non-pathogenic
Malignant PT	*SF3B1*	p.Gly83Gly	synonymous_variant	8.80%	385	No LOH	0.55	0.0006	0.3952	Subclonal	190	2	198285804	T	C	.	.	.		TRUE	TRUE	TRUE	Non-pathogenic
Malignant PT	*TERT*	c.-124C>T	upstream_gene_variant	27.90%	181	No LOH	1.00	0.9388	0.8784	Clonal	158	5	1295228	G	A	.	.	.					Likely pathogenic
