# Supplementary material for: Massively parallel sequencing analysis of synchronous fibroepithelial lesions supports the concept of progression from fibroadenoma to phyllodes tumor
Source: NPJ Breast Cancer. 2016 Nov 16;2:16035–. doi: 10.1038/npjbcancer.2016.35 (PMC5515337; doi:10.1038/npjbcancer.2016.35)
Supplement: Supplementary Table 4 [file npjbcancer201635-s8.pdf]

**Supplementary Table 4: List of somatic mutations found in the fibroepithelial lesions of the breast validated using amplicon resequencing.**

| Sample ID | Gene | Amino Acid Change | Effect | Depth | Chromosome | Position | Reference Allele | Alternate Allele |
|---|---|---|---|---|---|---|---|---|
| Benign PT | *MED12* | p.Gly44Val | missense_variant | 301248 | X | 70339254 | G | T |
| Fibroadenoma 1 | *MED12* | p.Gly44Cys | missense_variant | 387904 | X | 70339253 | G | T |
| Fibroadenoma 2 | *MED12* | p.Gly44Val | missense_variant | 333425 | X | 70339254 | G | T |
| Fibroadenoma 3 | *MED12* | p.Gly44Asp | missense_variant | 289436 | X | 70339254 | G | A |
| Malignant PT | *MED12* | p.Gly44Asp | missense_variant | 307470 | X | 70339254 | G | A |
| Malignant PT | *TERT* | c.-124C>T | upstream_gene_variant | 117714 | 5 | 1295228 | G | A |
