# Supplementary material for: Massively parallel sequencing analysis of synchronous fibroepithelial lesions supports the concept of progression from fibroadenoma to phyllodes tumor
Source: NPJ Breast Cancer. 2016 Nov 16;2:16035–. doi: 10.1038/npjbcancer.2016.35 (PMC5515337; doi:10.1038/npjbcancer.2016.35)
Supplement: Supplementary Table 5 [file npjbcancer201635-s9.pdf]

Supplementary Table 5: Analysis of clonal relatedness based on MED12 mutation data

		Lim et al (fibroadenomas)	Tan et al (phyllodes tumors)	Lim et al and Tan et al (fibroadenomas and phyllodes tumors)	Piscuoglio et al (fibroadenomas)	Piscuoglio et al (phyllodes tumors)	Piscuoglio et al (fibroadenomas and phyllodes tumors)	Piscuoglio et al (fibroadenomas, excluding the cases from the current cohort)	Piscuoglio et al (phyllodes tumors, excluding the cases from the current cohort)	Piscuoglio et al (fibroadenomas and phyllodes tumors, excluding the cases from the current cohort)
**Total number in cohort**		98	79	177	100	76	176	97	74	171
**cases with c.131G>T (G44V)**	**n**	3	11	14	8	5	13	7	4	13
	**%**	3.1%	13.9%	7.9%	8.0%	6.6%	7.4%	7.2%	5.4%	7.6%
**cases with c.131G>A (G44D)**	**n**	20	9	29	28	9	37	27	8	37
	**%**	20.4%	11.4%	16.4%	28.0%	11.8%	21.0%	27.8%	10.8%	21.6%
**Probability of two unrelated samples sharing**	**c.131G>T (G44V)**	0.0009	0.0194	0.0063	0.0064	0.0043	0.0055	0.0052	0.0029	0.0058
	**c.131G>A (G44D)**	0.0416	0.0130	0.0268	0.0784	0.0140	0.0442	0.0775	0.0117	0.0468
