# Supplementary material for: Massively parallel sequencing analysis of synchronous fibroepithelial lesions supports the concept of progression from fibroadenoma to phyllodes tumor
Source: NPJ Breast Cancer. 2016 Nov 16;2:16035–. doi: 10.1038/npjbcancer.2016.35 (PMC5515337; doi:10.1038/npjbcancer.2016.35)
Supplement: Supplementary Table 6 [file npjbcancer201635-s10.pdf]

Supplementary Table 6: List of primers used for *FLNA* Sanger sequencing

Exon	Forward	Reverse	Amplicon lenght
**ex 2**	CGCTTCGGGGCGTCCCTCAC	CCGGCTTCTCGCGCCTCAAAAT	413
**ex 3**	CTGCCTCTGCGCCCCCTC	GGCCACTCTTCTACTCACAGACAGC	287
**ex 4**	TCCCCTACAGCTGTAGCCAG	CGGCCCCCAGACTCACAG	168
**ex 5**	GGCACACGGGTGCACCCCT	GAGGCCCGATGGCTCCTGTT	218
**ex 6**	GCTGCGGGGCCTCTGCTG	AGCTGGCCCGTGGTTGGCT	189
**ex 7**	GAGGTCACAAGCCTCCCC	CTTAGCAAAGGCTCACAGGC	147
**ex 8**	CACAGACCAGCTGGGCCTT	AGCCCTACCCTCACGGCC	233
**ex 9**	GTGCCACAGCAGAGGGCAGT	TGGGGCCTGGGAGCTGAG	269
**ex 10**	GCCACCCATCCTGGCCTG	AGCTGCTGGTGCTCAGCTG	208
**ex 11**	TGCAGAGCTGGGAGAGGGAT	GCTGGGCAGGCCTGAGGC	194
**ex 12**	GTCCCCAGGTGCCCATGCT	CCTCGGTGTTCCCTGTGTGG	207
**ex 13**	GGGCGAGACTTAGGCCATCA	TCCAGGGCACTGAGGGGACT	263
**ex 14**	ACCTGCCCTGCCCCCAAC	CCTGGCCCCCTGACAGCT	184
**ex 15**	CGAGATGGACTAAAGGCCGG	GACCAGCAGGCCACCTGCT	214
**ex 16**	AGCCACCTGCCCTCCCAC	GCTCATGCCCTTGCCCTT	194
**ex 17**	AATGACATCTTAGCGGCCAG	CGAGGTCTCAGCCTCCGC	231
**ex 18**	TGCCCCGCAACCTGCCAT	CCTCCTCTAAGGCCTTCTCCTCCC	161
**ex 19**	GGGGACTCGGTGACTGTAGT	AGGGTGCCCCTACATGGT	240
**ex 20**	CAAAGGCCTTTGCGACAA	TCTAAATACCCCCTTCCCTTC	188
**ex 21**	GGTTCTACCCTTAGGGCCTC	TCATGTCTCTCTCCTTCCTTTCA	333
**ex 22_1**	AATTGGCCCACCTCCCCA	GTGTGGCCTTGGCTCCTGAC	370
**ex 22_2**	TCCCCCGTCACATACCCC	TTGACGCATCCAAAGTCAAG	368
**ex 23**	CCGTCTGCCAGCCTGTGGGA	CCCGAGCCCCAAGCCTCCT	244
**ex 24**	CCACCAGCCACACGGGCT	CCGGGCTCCTCCTGACCTG	233
**ex 25**	CAGTCCCAGCCCTGCCCT	AACCCCAGGAATGACCGGCT	231
**ex 26**	TCCTGACCCCTGGCTCCA	TCATACAGCCGCCATCCCTT	241
**ex 27**	GCCACTCCCCACAGGCAG	ACTAGCTGATGCTCTGTCCCTG	194
**ex 28**	CTCCCCTGCCTGTGCCCG	CCTGTCTGACTGCTCACAACACCA	213
**ex 29**	CGAGCGCCGCAGCGGCCAAC	TTGGGCCAACTGCCTCCCCTGC	260
**ex 30**	ACACCCCTGCTGACCTACCCC	TGGCCCCCCTTTGGGGAC	318
**ex 31**	GCTAGCCCCAGTGTCCCTA	CAGACTCTCCAGCAGCTCTCT	166
**ex 32**	TTGGCTCCCGAGCTCCTT	CTGTCCCTCACCCATGCC	177
**ex 33**	TGGCGGTGGAGTGGGCAG	TGAGCTGCCCTGACCTCAGC	211
**ex 34**	GCACAGAGCAGGTCAAGACC	GCTGCCATTCCACAAGGC	198
**ex 35**	CACCCGTTTCTGTCACTGCT	GCATAGTTCCCATGCTCACC	244
**ex 36**	CCTTTCTGAACCCCCTGGAC	TGGACGCACACTGATGGCT	232
**ex 37**	GCTCCCGCCCCAGCTGGT	GCCCTCCCTGACTGACAGCC	274
**ex 38**	AGTGAGGGGGGCTGCCGA	CATAGCACCGAGGCTCAGGG	223
**ex 39**	CCAGGACCCCTCCCCAGGCT	ACTGGAGGGGCGTGGGCC	192
**ex 40**	AGGAGAGCGAGCACTCGG	GGGCTGCTGCTCACTAGC	337
**ex 41**	CCCACCTCCTCCAACCCC	GCAGCCTTCAGTGAGGACAA	208
**ex 42**	GAGCCCCAGGTGGGCGGTTT	CTGATGAGCCGGTCTTACACTCTTTCC	186
**ex 43**	TTCCAGCCAGCAGGGCAG	GCACCCCCATCTAACCATGT	212
**ex 44**	TGTGTGCACACGTGCAGCC	GCCCCAGGCCCACAGCAT	246
**ex 45**	TTGGGCAGATGCCAATAGCT	CCAGTCTGGCTCTGCCTGA	288
**ex 46**	GGTGGGAAGGTGGGCCGG	AGCCACCTCTTAGCCCCACCC	274
**ex 47**	GGCTTGGGGGCTGCCGGCT	CTGGGACCTGGGACTGAGGACCC	258
